# The Influence of Crossbreeding on the Composition of Protein and Fat Fractions in Milk: A Comparison Between Purebred Polish Holstein Friesian and Polish Holstein Friesian × Swedish Red Cows

**DOI:** 10.3390/nu16213634

**Published:** 2024-10-25

**Authors:** Paweł Solarczyk, Jan Slósarz, Marcin Gołębiewski, Antonio Natalello, Martino Musati, Giuseppe Luciano, Alessandro Priolo, Kamila Puppel

**Affiliations:** 1 Department of Animal Breeding, Institute of Animal Sciences, Warsaw University of Life Sciences, 02-786 Warsaw, Poland; 2 Department of Agriculture, Food and Environment, University of Catania, 95123 Catania, Italy

**Keywords:** milk, protein, fat, vitamins, purebred, crossbreed

## Abstract

Background/Objectives: In this study, the differences in protein and fat bioactive components between the milk from purebred Polish Holstein Friesian (PHF) cows and PHF cows crossbred with Swedish Red (SRB) were investigated. The objective was to assess the impact of genetic variation on the nutritional quality of their milk. Methods: This study was conducted at the Warsaw University of Life Sciences’ (WULS) experimental dairy farm in Warsaw, Poland, and involved 60 primiparous cows divided into two groups: 30 PHF×SRB crossbred cows and 30 purebred PHF cows. All cows were housed in a free-stall system with an average lactation yield exceeding 10,000 kg/lactation. The milk composition analyses included total protein, casein, whey protein, fatty acid profiles, and vitamin content. Results: Milk from the PHF×SRB hybrids showed a significantly greater total protein content (3.53%) compared to that from the purebred PHF cows (3.28%). The casein content was higher in the hybrids’ milk (2.90%) than the purebreds’ milk (2.78%), while the whey protein levels were lower in the purebred milk (0.50%) than in the hybrid milk (0.63%). The hybrids exhibited higher concentrations of certain saturated fatty acids in their milk, while the purebreds’ milk contained greater amounts of beneficial unsaturated fatty acids and fat-soluble vitamins—E, D, and K. Conclusions: These results indicate that genetic selection through crossbreeding can enhance the nutritional quality of milk. The differences observed in protein, fatty-acid, and vitamin content underscore the role of the genotype in milk composition, suggesting that breeding strategies can optimize dairy products’ health benefits.

## 1. Introduction

Milk is recognized as an excellent food and a source of energy and high-quality protein, vitamins, and minerals in a digestible form available to humans, especially during the growing period [1]. Cow’s milk is the most widely used in human nutrition and accounts for about 81% of world production [2].

The protein in cow’s milk has high biological value and, importantly, has high digestibility [3]. Protein undergoes hydrolysis in the gastrointestinal tract, resulting in the formation of peptides that exhibit bioactive properties [4]. Milk protein is composed of two main fractions, namely, casein (C) (approximately 80%) and whey protein (WP) (approximately 20%) [3,5]. Although the WP content of milk is relatively low, WP has the highest biological value among the proteins consumed by humans [6].

The casein fraction is mainly responsible for the capacity to process milk in dairy production and, after consumption, is involved in regulating metabolism and activating antioxidant enzymes, as well as being a source of trace elements [7].

The main WP in ruminant milk is β-lactoglobulin (BLG), which is synthesized in the mammary glands. BLG binds to and transfers hydrophobic compounds such as retinol, vitamin D2, cholesterol, and fatty acids (FAs), thus improving their bioavailability. BLG is involved in passive immunity in newborns and in the regulation of phosphorus metabolism in the mammary glands [8,9,10]. BLG binds with free fatty acids (FFAs), altering the digestion of milk fat by increasing lipase activity [11,12]. It has the ability to adhere to microorganisms, preventing pathogen colonization, inhibits viral replication, and has anti-cancer effects [13,14]. The second WP in milk is α-lactalbumin (ALA), which is produced in the epithelial cells of the mammary glands and is involved in the biosynthesis of lactose and milk [15]. ALA is recognized as a major source of essential amino acids (EAAs) [16]. ALA has the ability to bind and transport ions: calcium, magnesium, sodium, potassium, zinc, and manganese [17,18]. It has anti-cancer properties, aids in mineral absorption, has immunomodulatory effects, and prevents damage to the gastric mucosa [18,19]. BLG and ALA are considered milk allergens [20].

Another WP is lactoferrin (Lf), which is responsible for chelating iron, suppressing the inflammatory response, and inhibiting oxidative stress. The action of Lf is dependent on iron saturation [21]. It has antimicrobial, antifungal, antiviral, anti-inflammatory, and anti-cancer properties; these are mainly related to its ability to alter the permeability of cell membranes, thus contributing to the lysis and death of cells considered harmful [22,23,24]. Yet another WP is bovine serum albumin (BSA). This is a protein derived from blood serum and enters the milk via secretory cells. BSA has a high content of EAAs; it transports long-chain fatty acids and steroid hormones and binds metal ions and aromatic compounds [18]. Lactoperoxidase (Lp) is a WP secreted into the secretory cells of the mammary gland [25]. Lp is responsible for producing an effective immune response to inflammation [26], fights against microorganisms, and is used in the protection of the mammary glands and the digestive tract of newborns [27,28,29]. It has anti-cancer and antioxidant properties and is also used to preserve food products [30,31,32]. Lysozyme (Lz) is a WP with the following properties: antibacterial, antiviral, antifungal, anti-inflammatory, with antihistamine and anti-tumor activities. Additionally, it demonstrates immunostimulant properties [33,34]. Studies have shown that Lz also has the potential to act synergistically with antibiotics [35].

The fat fraction of milk also plays an important role in the human diet. It is commonly believed that the consumption of C12:0, C14:0, and C16:0 acids has an adverse effect on the human body and is related to an increased number of low-density lipoproteins (LDLs) and the occurrence of atherosclerosis and coronary artery disease; however, the other saturated fatty acids (SFAs) found in milk have a neutralizing effect on these acids by increasing the concentration of high-density lipoproteins (HDLs) [36,37,38]. In addition, studies on the effects of C12:0, C14:0, and C16:0 acids indicate that, due to the presence of calcium, peptides, and phosphorus, these acids are modified in a manner that restricts their harmful properties [39,40]. From a consumer’s perspective, butyric acid (C4:0–4.4%), which belongs to the SFA group (of which milk is almost the only source), plays an important role in that it has been shown to have anti-cancer properties [41] and is an essential source of energy for the intestines, maintaining homeostasis, preserving the function of the mucous membrane, and ensuring defense against pathogens [38]. The second fraction of fatty acids is unsaturated fatty acids. In this group, the highest content found is that of oleic acid (C18:1 c9–24–35%), the effect of which is related to C18:0. Both of these acids show anti-carbohydrate properties and have positive effects on human health [38]. The third fraction is polyunsaturated fatty acids (PUFAs). A particular role is played here by conjugated linoleic acid (CLA) and its isomers, formed by microbial biohydrogenation in the digestive tract of ruminants. CLA exhibits a cardiovascular immune function and has anti-cancer hypolipidemic, anti-atherosclerosis, and anti-diabetic properties [38,42,43]. The main source of CLA in the human diet is milk, but CLA is also present in ruminant meat [44,45].

Milk is also a source of vitamins. The content of fat-soluble vitamins depends on the content of fat in the milk. Vitamin A is particularly important during both human and animal growth periods as it is responsible for the body’s normal development and immune activity by supporting epithelial barrier function. It is considered an important vitamin for maintaining normal vision and is involved in inhibiting the spread of cancer through its antioxidant properties [46,47]. Vitamin D is responsible for the control of calcium homeostasis in the body and influences the inhibition of cell proliferation and apoptosis, which can enhance immunity. It also has an inhibitory effect on the growth of tumors, especially skin tumors [47,48]. Vitamin E is primarily responsible for inhibiting the clumping of platelets, and it induces the vasodilation of blood vessels. It exhibits antioxidant properties through trapping and inhibiting the production of reactive oxygen species. The antioxidant function depends on the presence of other vitamins (C and B) as well as selenium and glutathione. Vitamin D is also responsible for preventing the skin aging process and the formation of scars by protecting against oxidative stress in the skin [47,49]. Vitamin K is responsible for blood clotting, normal bone development, and the inhibition of osteoporosis and cardiovascular disease. Due to its effect on blood coagulation, vitamin K accelerates the wound-healing process and reduces reactive oxygen species due to its antioxidant properties [47,50].

The aim of the present study was to determine whether crossbreeding influences the levels of bioactive compounds and antioxidant potential, both of which play a crucial role in the nutritional quality and oxidative stability of milk. The study hypothesis was that crossbreeding PHF×SRB cows enhances milk composition, particularly by increasing its antioxidant potential, leading to improved oxidative stability and overall nutritional quality.

## 2. Materials and Methods

The research was conducted following the ethical guidelines of the Second Ethics Committee for Animal Experimentation in Warsaw, Ministry of Science and Higher Education (Warsaw, Poland), which approved all procedures (permission no. 10/2011). All cows involved in the study were handled according to the regulations of the Polish Council on Animal Care. The experimental design and procedures were also approved by the Warsaw University of Life Sciences Care Committee. This oversight ensured compliance with ethical standards in animal research.

### 2.1. Animals and Sampling

The experimental study was conducted at a dairy farm situated on the premises of Warsaw University of Life Sciences (WULS) in Warsaw, Poland. This facility houses approximately 350 cows in a free-stall housing system and boasts an average lactation yield that exceeds 10,000 kg of milk. Within the framework of this investigation, a meticulously selected group of 60 primiparous cows underwent a thorough selection process, leading to their categorization into two distinct groups. The experimental group comprised 30 crossbred cows, identified as Polish Holstein Friesian × Swedish Red (PHF×SRB), while the control group encompassed 30 purebred Polish Holstein Friesian (PHF) cows.

The dietary regimen was formulated based on the recommendations provided by the INRA system. Administered ad libitum, the diet consisted of a total mixed ration (TMR) composed of various components, including maize silage (12.10 kg/d DM), alfalfa silage (4.80 kg/d DM), corn silage (2.00 kg/d DM), soybean meal (2.50 kg/d DM), pasture ground chalk (0.20 kg/d DM), salt (0.05 kg/d DM), rapeseed meal (1.50 kg/d DM), and magnesium oxide (0.06 kg/d DM). Notable nutritional parameters pertaining to the TMR included total kilograms of dry matter (23.10) and daily intake (19.90 kg).

Milk sampling was carried out 10 times at 30-day intervals between 10 and 280 ± 5 days postpartum. Individual milk samples, each measuring 250 mL, were obtained during both morning and evening milking sessions. Subsequently, these samples were diligently preserved in sterile containers and expeditiously transported to WULS’s Milk Testing Laboratory for in-depth compositional analysis. All analyses were performed in duplicate five times.

Strict criteria were implemented to select cows for the study, ensuring that only animals free from hoof issues, such as sole ulcers, and other health conditions that may have influenced the data were included. All cows were under veterinary care. There were no metabolic disorders, as indicated by the measurements of non-esterified fatty acids (NEFAs), β-hydroxybutyric acid (BHBA), glucose, and F/P (fat/protein) ratio for the cows all having the correct values. Additionally, somatic cell count (SCC) levels did not indicate mastitis.

### 2.2. Chemical Analyses

The assessment of the basic milk parameters, specifically the fat, protein, lactose, and casein contents, was conducted by employing an automated infrared analysis methodology facilitated by a MilkoScan FT 120 analyzer (Foss Electric, Hillerød, Denmark).

The trans-esterification method outlined in EN ISO 12966-2:2017 [51] was employed for the methylation of fatty acids. Identification of individual fatty acids within the crude fat samples was undertaken using an Agilent 7890A GC system (Agilent, Waldbronn, Germany), following the methodology established by Puppel et al. [52]. The identification process was substantiated using pure methyl ester standards, including FAME Mix RM–6 (Lot LB 68242), Supelco 37 Comp. The FAME Mix (Lot LB 68887), methyl linoleate (Lot 094K1497), and conjugated CLA (9Z, 11E) (Lot BCBV3726) were all sourced from Supelco (Bellefonte, PA, USA).

The concentrations of α-lactalbumin, β-lactoglobulin, lysozyme, lactoferrin, bovine serum albumin, and lactoperoxidase were determined using an Agilent 1100 Series RP-HPLC device (Agilent Technologies, Waldbronn, Germany) according to the methodology described by Puppel et al. [53]. All samples were analyzed in duplicate. The total run time was 44 min, the flow rate was 1.2 mL min^−1^, and the detection wavelength was 220 nm. The injection volume of the final solution was 25 μL. The identification of the peaks corresponding to α-lactalbumin, β-lactoglobulin, lysozyme, lactoferrin, bovine serum albumin, and lactoperoxidase was confirmed via comparison with standards (Sigma–Aldrich, St. Louis, MO, USA).

The total antioxidant status in blood plasma was measured using a NanoQuant Infinite M200 Pro analyzer (Tecan Austria GmbH, Grödig, Austria) with ELISA kits from Randox Laboratories (Crumlin, UK), specifically the Total Antioxidant Status kit (Cat. No. NX2331).

The concentrations of α-tocopherol (vitamin E), α-retinol (vitamin A), vitamin D, vitamin K, and β-carotene were determined via reversed-phase high-performance liquid chromatography (RP-HPLC) using an Agilent 1100 Series system (Agilent Technologies, Waldbronn, Germany) according to the method of Solarczyk et al. [44]. Chromatographic separations were performed at ambient temperature on a ZORBAX Eclipse XDB column (Agilent Technologies, Waldbronn, Germany) under solvent gradient conditions. The mobile phase consisted of methanol (Merck, Darmstadt, Germany) and water (Sigma–Aldrich, St. Louis, MO, USA) in a 950:50 (*v*/*v*) ratio, with a flow rate of 1.0 mL/min. Detection was carried out at a wavelength of 280 nm, with an injection volume of 25 μL. All samples were analyzed in duplicate, and peak identification was verified via comparison with standards from Sigma–Aldrich (St. Louis, MO, USA).

### 2.3. Statistical Analysis

The data underwent a comprehensive statistical compilation employing an analysis of variance (ANOVA) through the least-squares method facilitated by PS IMAGO PRO 10.0 software [54]. Significant differences among group means were determined using the F-statistic. The distribution characteristics of the whey protein, fatty acid, and vitamin composition were examined through the application of the Shapiro–Wilk test.

## 3. Results

The milk analyzed in this experiment was from cows that had no mammary gland inflammation problems or metabolic diseases, as evidenced by the results in Table 1, where the SCC for the purebred PHF cows is 116 × 10^3^/mL, and that of the PHF×SRB hybrids is 123 × 10^3^/mL; the EU standard for this parameter is 400 × 10^3^/mL [55]. In addition, the cows were under constant veterinary care—lameness, diarrhea, and fever were absent.

### 3.1. Protein Fraction in the Milk

Table 2 illustrates the influence of the cow genotype on the composition of casein and whey proteins in the milk. The total protein content in the milk from the PHF×SRB crossbred cows was approximately 7.62% higher than that of the purebred PHF cows. In the purebred PHF milk, C constituted 84.76% of the total protein, whereas in the crossbred cows, it accounted for 82.15%, indicating that the C content was about 4.32% greater in the crossbred cows’ milk. In contrast, WP concentrations were approximately 26% lower in the purebred PHF milk compared to those in crossbred cows’ milk. This difference can be attributed to the genetic predisposition of the HF breed, recognized for its high milk production resulting from a dilution effect, along with the genetic contributions of the Swedish Red breed to the overall milk composition. Furthermore, Lz concentrations were approximately 17.19% lower in the crossbred cows’ milk than in purebred PHF milk. Lf levels showed a more pronounced reduction, being about 55.26% lower in the crossbred cows’ milk. Conversely, the BSA content was approximately 27.78% higher in the crossbred cows’ milk compared to the purebred PHF milk. Additionally, the levels of BLG in the crossbred cows’ milk were approximately 45% higher than those in the purebred PHF cows. Finally, Lp activity was about 14.71% higher in the crossbred cows’ milk compared to purebred PHF milk.

### 3.2. Fat Fraction in the Milk

#### 3.2.1. Fatty Acids

The analysis of selected SFAs (Table 3) in the milk of purebred PHF cows and PHF×SRB crossbred cows revealed genotype-dependent variations. The crossbred hybrids demonstrated higher concentrations of short- and medium-chain SFAs, including butyric acid (C4:0), capric acid (C10:0), lauric acid (C12:0), and myristic acid (C14:0), when compared to the purebred PHF cows. These differences were statistically significant, indicating a genotype-driven improvement in the synthesis of these specific SFAs. In contrast, the PHF cows had a higher content of stearic acid (C18:0), suggesting that divergent metabolic pathways affect long-chain fatty acid synthesis. The observed disparities suggest that crossbreeding selectively influences the milk’s FA profile, enhancing certain FAs associated with different nutritional and functional properties.

The comparison between the unsaturated fatty acid (UFA) content in the purebred PHF cows and that in the PHF×SRB crossbred cows revealed notable genotype-related differences. The crossbred hybrids exhibited a higher concentration of palmitoleic acid (C16:1), while the PHF cows showed significantly higher levels of C18:1 c9 and vaccenic acid (C18:1 t11). Furthermore, the PUFA content was greater in the PHF milk, with higher levels of linoleic acid (C18:2 c9,c12 *n*-6), α-linolenic acid (C18:3 *n*-3), and CLA, suggesting a more favorable UFA profile for the purebred cows. Conversely, the crossbred hybrids displayed elevated levels of γ-linolenic acid (C18:3 *n*-6) and certain long-chain *n*-6 fatty acids, indicating a differential FA metabolism between the two genotypes. While differences in trans FAs were not statistically significant, the overall findings suggest that the genotype significantly influenced the composition of UFA, with potential implications for the nutritional and functional properties of milk.

#### 3.2.2. TAS and Lipophilic Vitamins

Table 4 presents the effect of genotypes on the total antioxidant status (TAS) and the lipophilic vitamin content in the milk. The TAS was significantly higher in the milk of the purebred PHF cows compared to that of the PHF×SRB crossbred cows, reflecting an approximately 14.0% greater antioxidant capacity in the purebred PHF milk. The concentration of vitamin E was substantially greater in PHF cows than in the crossbred cows, with a difference of approximately 28.3% in favor of the PHF cows. A similar trend was observed for vitamins D and K; specifically, the vitamin D content was about 15.3% higher in PHF cows, while vitamin K levels were approximately 10.1% higher. In contrast, no significant differences were noted in the concentrations of β-carotene or vitamin A between the PHF cows and the crossbred cows, suggesting that these specific vitamins may not be influenced by the genetic factors associated with crossbreeding. These findings emphasize a significant genetic influence on the milk’s antioxidant status and vitamin profile.

## 4. Discussion

### 4.1. Protein Fraction in the Milk

The WP fraction is the main source of the so-called EAAs that are responsible for maintaining adequate homeostasis in the body [56]. These amino acids include sulfur-containing amino acids (SAAs), which are responsible for specific immune responses, reducing oxidative stress, and protecting against cancer [57]. They also include branched-chain amino acids (BCAAs), which are involved in blood glucose metabolism and homeostasis, as well as fat metabolism and regulating the synthesis of skeletal muscle tissue protein [58,59,60]. WPs are also involved in antioxidant activity, which is determined primarily according to the histidine and hydrophobic amino-acid content [5]. The role of WPs in the antioxidant process is to scavenge free radicals, chelate metals, and recover the thiol–SH group in proteins [7]. The results of this study reveal significant differences in the protein composition and bioactive components of the milk from purebred PHF cows compared to the PHF×SRB crossbred cows. These findings are crucial for understanding how crossbreeding influences milk quality and have direct implications for dairy production. The demand for milk and dairy products, which is driven by their nutritional value, is highlighted by Górska-Warsewicz et al. [61], who noted that milk proteins constitute a substantial portion of daily nutrient supply. The observed differences in individual protein fractions between PHF cows and PHF×SRB crossbred hybrids stem from complex interactions involving genetic and physiological factors. The total protein content was notably higher in the crossbred cows compared to the purebreds, which is advantageous for dairy production, as elevated protein levels enhance the nutritional quality of milk, rendering it more suitable for cheese production. The C content also exhibited significantly higher levels in the crossbred cows relative to the purebreds. As a primary component of milk, C is essential to cheese-making processes. Interestingly, Lindmark-Mansson et al. [62] reported shifts in the ratios of C to WP in Swedish cows, observing a decrease in the C content alongside increased WP, which they attributed to dairy cow breeding programs. Similarly, Puppel et al. [63] noted a trend toward lower casein levels in hybrid cows of Scandinavian Red descent. Furthermore, Gustavsson et al. [64] indicated that the SRB breed is characterized by a higher overall protein content in its milk compared to HF, suggesting that the genetic influence of the SRB breed may contribute to the enhanced protein profile observed in the PHF×SRB hybrid cows in this study.

The results indicate that both Lz and Lf levels were significantly lower in the crossbred cows compared to the purebred PHF cows. Lz is a glycoprotein found in milk that has antibacterial properties. It directly contributes to the innate immune defense of the milk by breaking down the peptidoglycan layer of bacterial cell walls. This action helps maintain the microbial stability of the milk [65]. Lf, another important glycoprotein, exhibits antibacterial activity and plays a crucial role in regulating iron homeostasis and modulating the immune response [66]. The observed lower Lz and Lf levels in the crossbred cows may be surprising given the expectation of heterosis associated with crossbreeding, which often leads to enhanced performance traits and increased resilience; however, the lower levels of these proteins may indicate that the specific genetic combinations in the crossbred population do not favor the expression of these immune-related proteins. This suggests that the crossbreeding strategy might influence not only milk production traits but also the immunological profile of the milk. The diminished presence of lysozyme and lactoferrin in the milk of the crossbred cows may reflect a complex interaction between the genetic backgrounds of the breeds involved and their respective immune response mechanisms [63]. While heterosis can enhance various traits, the findings in this study indicate that it does not uniformly translate into improved levels for all immune components.

Unlike the above proteins, the levels of ALA did not exhibit significant differences between the purebred and crossbred groups, suggesting that the genetic background of the cows did not influence the expression of this protein. ALA, which is primarily involved in lactose synthesis, plays a critical role in the milk’s composition, but the stable levels across both groups indicate consistent production mechanisms. BSA concentrations were significantly higher in the crossbred cows compared to the purebred cows. The higher BSA level is particularly noteworthy, as BSA functions as a carrier protein, facilitating the transport of fatty acids, hormones, and other bioactive compounds, thereby enhancing the nutritional profile of the milk. Increased BSA levels may contribute to improved health outcomes for consumers due to the potential role of this protein in binding and transporting bioactive substances. Similarly, BLG levels were significantly elevated in the crossbred cows. The greater concentration of BLG, a predominant whey protein, is associated with enhanced functional properties, including emulsification, foaming, and gelation. These characteristics are essential for the processing and texture of various dairy products, which may be advantageous for both producers and consumers. Research, including findings by Gustavsson et al. [64], suggests that a higher BLG content could be linked to specific genetic variants, implying that breeding strategies could be designed to select for these beneficial traits. Lp levels were also significantly higher in the crossbred cows. Lp is known for its antimicrobial activity, which contributes to the preservation of milk by inhibiting the growth of pathogenic bacteria [67]. The elevated levels of Lp in the crossbred cows’ milk may enhance the microbial quality and extend the shelf life of dairy products.

The increased concentrations of key whey proteins (BSA, BLG, and Lp) in the milk from the crossbred hybrids may be indicative of a more robust immune response in these animals. Enhanced immune activity could lead to better protection of the mammary gland against pathogenic intrusions, which would correlate with the observed lower somatic cell count in the milk of the crossbred hybrids. A lower SCC is typically associated with improved udder health and reduced mastitis incidence, further substantiating the role of genetic factors in influencing both milk composition and quality [68]. Furthermore, elevated levels of these whey proteins not only enhance cow health but also augment the biological activity of the milk. The proteins possess various bioactive properties that may confer health benefits upon consumers, establishing their significance in functional food applications. Notably, the exceptional heat stability of these milk proteins during thermal processing allows them to retain their functional properties, thereby improving the quality of the final dairy product [69]. Interestingly, the findings of Maurmayr et al. [70] indicate that while their hybrids displayed higher ALA concentrations, they exhibited lower BLG levels. This variability underscores the complexity of genetic factors influencing protein expression across different crossbreeding scenarios, highlighting the necessity for further investigation into the specific genetic determinants that affect whey protein profiles in dairy cattle.

### 4.2. Fat Fraction in the Milk

#### 4.2.1. Fatty Acids

Milk fat is a crucial component in dairy products, significantly influencing their nutritional quality, sensory properties (taste and aroma), and functional applications in food processing [71]. The fat in milk is predominantly composed of triacylglycerols (TAGs). The remaining components include diacylglycerols, cholesterol, phospholipids, and FFAs. The complexity of TAGs arises from their diverse composition, which includes fatty acids, each exhibiting unique physicochemical and bioactive properties. The specific composition of these FAs is influenced by various factors, such as dietary intake, microbial fermentation in the rumen, de novo synthesis in the mammary gland, and the physiological state and age of the animal [37,52,53,72,73,74,75]. This study reveals significant differences between the fatty acid profiles of the purebred PHF cows and the PHF×SRB crossbred cows. These variations can be attributed to genetic differences resulting in distinct metabolic pathways and FA synthesis mechanisms.

A critical factor influencing milk fat production is the cow’s energy balance. The energy necessary for milk synthesis is derived from dietary sources, while energy deficits are compensated for by mobilizing adipose tissue [76]. The PHF breed is known for its high productivity and elevated energy requirements, which may not always be met through feed intake alone. Consequently, PHF cows often mobilize body reserves more extensively than SRB cows, which have been selectively bred for improved feed efficiency and better metabolic regulation [77]. In this study, it was observed that the PHF cows exhibited higher concentrations of certain FAs, such as stearic acid (C18:0) and C18:1 c9. Elevated levels of these FAs can indicate a state of negative energy balance, highlighting the physiological implications of milk production under conditions of varying energy availability.

The current analysis indicates that PHF×SRB crossbred hybrids had higher concentrations of short- and medium-chain fatty acids compared to the purebred PHF cows. Conversely, the PHF cows exhibited significantly higher levels of beneficial long-chain fatty acids. These findings are consistent with the existing literature, which suggests that the FA composition of milk is influenced by genetic selection and metabolic efficiency. Higher levels of specific FAs in hybrids may be linked to their metabolic pathways, which differ from those of PHF cows. While certain FAs are often associated with less favorable health outcomes, the presence of beneficial long-chain fatty acids in PHF cows suggests potential health-promoting properties. The role of *n*-3 PUFAs is particularly noteworthy due to their health benefits, including anti-inflammatory properties and cardiovascular protection [78]. Essential FAs, such as linoleic acid and α-linolenic acid (ALA) are vital for human health as they cannot be synthesized endogenously [78]. This study indicates that CLA levels were higher in milk from the PHF cows, correlating with elevated ALA content. This suggests that selective breeding strategies could enhance the presence of these beneficial FAs in milk.

The observed differences in FA profiles between PHF and crossbred cows have significant implications for dairy production and consumer health. Regarding fat content, the crossbred hybrids had higher levels of SFAs, specifically C4:0, C10:0, C12:0, and C14:0, compared to the purebred PHF cows. In contrast, the purebred PHF cows exhibited higher concentrations of beneficial UFAs, such as C18:0 and C18:1 c9. These findings indicate that the genotype significantly affects the FA profile of milk. The higher levels of beneficial long-chain fatty acids in milk from the PHF cows indicate that this breed may contribute positively to the nutritional quality of dairy products. Additionally, the increased concentrations of short- and medium-chain fatty acids in hybrids could influence their utility in various dairy applications, such as cheese production and other dairy products. These findings emphasize the importance of breeding programs focused on optimizing milk FA profiles. By selecting for desirable FA characteristics, dairy producers can enhance the nutritional value of milk, thereby meeting consumer demand for healthier dairy options.

#### 4.2.2. TAS and Lipophilic Vitamins

The results of this study demonstrate significant differences in vitamin content between the milk of the purebred PHF cows and the PHF×SRB cows. Specifically, milk from the PHF cows exhibited higher concentrations of vitamins E, D, and K compared to that of the hybrids. These disparities can be attributed to genetic factors and the metabolic pathways involved in vitamin synthesis and accumulation. Genes influence lipid metabolism significantly in dairy cows. This metabolism is crucial for the synthesis of fat-soluble vitamins. Enzymes, such as lipases, which are responsible for fat breakdown, can be genetically regulated, affecting the body’s ability to process lipids [79].

Vitamin E levels were markedly higher in the milk of the PHF cows compared to the hybrids. This difference may reflect the genetic capacity of PHF cows to synthesize or retain higher levels of this essential antioxidant, which plays a crucial role in maintaining cellular integrity and reducing oxidative stress [80]. The presence of vitamin E in milk is vital for both animal health and human nutrition, as it contributes to immune function and may have protective effects against chronic diseases [81].

A similar trend was observed with vitamin D, of which the PHF cows had higher concentrations compared to the hybrid cows. Vitamin D is critical for calcium metabolism and bone health, and its increased concentration in PHF milk may provide additional nutritional benefits. The genetic differences between the breeds may influence the cows’ ability to mobilize vitamin D and its subsequent accumulation in milk.

The findings showed that the PHF cows had a higher content of vitamin K than the hybrids. Vitamin K is essential for blood clotting and bone health, and the presence of greater amounts in the milk from PHF cows could enhance its functional properties. The observed differences in vitamin K content are likely influenced by genetic factors that affect the metabolism and storage of the vitamin within the mammary gland. Research indicates that the rumen’s fermentation process likely modulates the synthesis of the K and B vitamins, but comprehensive data on this topic remain scarce. Furthermore, studies have shown that the concentrations of vitamin K in milk often do not correlate well with cows’ dietary intakes. Haug et al. [82] suggest that this discrepancy may arise from the simultaneous processes of degradation and synthesis that occur in the rumen, which can affect the overall availability of these vitamins for absorption by the milk.

The observed difference in total antioxidant status between the purebred PHF cows and the PHF×SRB crossbred hybrids indicates a significant disparity in antioxidant capacity. The 14.09% greater TAS levels for the purebred PHF cows reflect their enhanced ability to combat oxidative stress, a critical factor in preserving milk quality. Higher TAS values are indicative of greater stability against oxidative degradation, thereby delaying lipid peroxidation and preserving the nutritional integrity of the milk. As noted by Puppel et al. [83], an elevated antioxidant potential is crucial for prolonging the delayed phase of protein oxidation and mitigating the formation of detrimental compounds such as dityrosine. The capacity to resist oxidative damage not only safeguards beneficial milk constituents but also extends the overall shelf life and stability of dairy products. This is particularly relevant in dairy production, where the preservation of sensory attributes and nutritional quality is essential for consumer acceptance.

Despite the fact that the same management practices were applied to both groups, the differences in vitamin content highlight the potential influence of genetics on vitamin synthesis and accumulation. Genetic factors may determine how efficiently each breed metabolizes vitamins, thus affecting their final concentrations in milk. This suggests that selective breeding for specific traits may improve the nutritional profile of milk produced by dairy cows.

## 5. Conclusions

This study reveals that there are significant differences between the bioactive components in milk from purebred Polish Holstein Friesian cows and that from Polish Holstein Friesian and Swedish Red crossbred cows. While the crossbred hybrids exhibited enhanced total protein content, the concentrations of beneficial unsaturated fatty acids, particularly CLA, and fat-soluble vitamins (E, D, and K) were markedly higher in the purebred PHF cows. These findings indicate that crossbreeding may improve specific traits without universally enhancing all aspects of milk quality. Moreover, the TAS values were significantly higher in the purebred PHF cows (1.70 mmol/L) compared to the hybrids (1.49 mmol/L). The elevated TAS values suggest a superior antioxidant capacity in PHF cows’ milk, which is crucial for delaying lipid peroxidation and maintaining nutritional integrity. The findings highlight the fact that genetic factors play a pivotal role in the synthesis and retention of vital nutrients and antioxidants in milk. In summary, the results underscore the necessity for targeted genetic selection in dairy cattle breeding programs, focusing on optimizing not only protein yields but also the nutritional quality of milk, including beneficial FAs, vitamins, and antioxidant capacity. These insights can guide breeding strategies in order to enhance the health benefits of dairy products, thereby aligning with increasing consumer demand for nutritionally superior milk.

## Figures and Tables

**Table 1:** Parameters for cows participating in the experiment.

	PHF (*n* = 30)		PHF×SRB (*n* = 30)		*p*-Value
	LSM	SEM	LSM	SEM	
DMP [kg]	30.08	0.146	27.45	0.149	0.000
Lactose [%]	5.06	0.009	4.79	0.009	0.000
SCC [10^3^/mL]	116	9.396	124	9.553	0.570
F/P	1.18	0.015	1.14	0.015	0.098
BHBA [mmol/L]	0.79	0.015	0.673	0.015	0.000
NEFA [mmol/L]	0.39	0.016	0.20	0.016	0.000
Glucose [mg/dL]	64.56	0.315	63.63	0.320	0.039

PHF—Polish Holstein Friesian cows, PHF×SRB—Polish Holstein Friesian and Swedish Red hybrid cows, DMP—daily milk production, SCC—somatic cell count, F/P—fat/protein ratio, BHBA—β-hydroxybutyric acid, NEFA—non-esterified fatty acids, LSM—least-squares mean, SEM—standard error of LSM.

**Table 2:** The influence of the cow’s genotype on the contents of individual proteins.

	PHF		PHF×SRB		*p*-Value
	LSM	SEM	LSM	SEM	
Protein [%]	3.28	0.016	3.53	0.016	0.000
Casein [%]	2.78	0.011	2.90	0.011	0.000
Whey protein [%]	0.50	0.030	0.63	0.042	0.000
Lz [μg/L]	20.18	0.510	16.59	0.518	0.000
Lf [μg/L]	0.38	0.023	0.17	0.024	0.000
ALA [g/L]	1.68	0.022	1.72	0.022	0.228
BSA [g/L]	0.18	0.005	0.23	0.005	0.000
BLG [g/L]	2.40	0.046	3.48	0.047	0.000
Lp [mg/L]	0.34	0.010	0.39	0.010	0.003

PHF—Polish Holstein Friesian cows, PHF×SRB—Polish Holstein Friesian and Swedish Red hybrid cows, Lz—lysozyme, Lf—lactoferrin, ALA—α-lactalbumin, BSA—bovine serum albumin, BLG—β-lactoglobulin, Lp—lactoperoxidase, LSM—least-squares mean, SEM—standard error of LSM.

**Table 3:** The influence of the cows’ genotypes on selected fatty acids.

	PHF		PHF×SRB		*p*-Value
	LSM	SEM	LSM	SEM	
Fat [%]	3.84	0.046	3.97	0.047	0.045
Selected fatty acid [g/100 g fat]					
SFA	64.50	0.193	64.12	0.196	0.161
C4:0	2.60	0.026	2.68	0.026	0.026
C6:0	1.53	0.017	1.47	0.018	0.017
C8:0	1.02	0.014	1.01	0.014	0.370
C10:0	2.05	0.034	2.39	0.034	0.000
C12:0	2.51	0.035	2.75	0.035	0.000
C14:0	8.99	0.068	9.27	0.069	0.003
C16:0	30.71	0.150	30.48	0.152	0.277
C18:0	12.19	0.089	11.16	0.091	0.000
C16:1 c9	1.63	0.015	1.71	0.016	0.001
C18:1 t11	2.69	0.043	2.76	0.044	0.279
C18:1 c9	25.25	0.168	23.77	0.171	0.000
C18:1 c11	1.24	0.009	1.17	0.009	0.000
PUFA	3.94	0.018	3.77	0.018	0.000
C18:2 c9,c12 *n*-6	2.16	0.014	2.04	0.014	0.000
C18:3 *n*-6	0.04	0.002	0.06	0.002	0.000
C18:3 *n*-3	0.34	0.003	0.32	0.003	0.000
C18:2 c9,t11	0.53	0.005	0.51	0.005	0.000
C18:2 t10,c12	0.03	0.001	0.02	0.001	0.000
C18:2 c9,t13	0.21	0.003	0.16	0.003	0.000
C20:2 *n*-6	0.02	0.001	0.04	0.001	0.000
C20:4 *n*-6	0.15	0.002	0.16	0.002	0.001
C20:5 *n*-3	0.10	0.002	0.08	0.002	0.000
C22:5 *n*-3	0.07	0.001	0.07	0.001	0.392
C22:6 *n*-3	0.01	0.001	0.02	0.001	0.000

PHF—Polish Holstein Friesian cows, PHF×SRB—Polish Holstein Friesian and Swedish Red hybrids, SFA—saturated fatty acid, PUFA—polyunsaturated fatty acid, LSM—least-squares mean, SEM—standard error of LSM.

**Table 4:** The influence of the cows’ genotypes on TAS and lipophilic vitamins.

	PHF		PHF×SRB		*p*-Value
	LSM	SEM	LSM	SEM	
TAS [mmol/L]	1.70	0.037	1.49	0.037	0.000
β-carotene [mg/L]	0.25	0.006	0.24	0.006	0.292
A [mg/L]	0.73	0.013	0.75	0.013	0.233
E [mg/L]	0.97	0.024	0.71	0.025	0.000
D [μg/L]	5.04	0.145	4.37	0.147	0.001
K [μg/L]	8.04	0.133	7.30	0.135	0.000

PHF—Polish Holstein Friesian cows, PHF×SRB—Polish Holstein Friesian and Swedish Red hybrids, TAS—total antioxidant status, LSM—least-squares mean, SEM—standard error of LSM.

## Data Availability

All data generated or analyzed during the study are included in this published article. The datasets used and/or analyzed in the current study are available from the corresponding author upon reasonable request.
